# Patient safety incident reporting software: A cross‐sectional survey of nurses and other users' perspectives

**DOI:** 10.1111/jan.16364

**Published:** 2024-08-11

**Authors:** Saija Koskiniemi, Tiina Syyrilä, Katri Hämeen‐Anttila, Santtu Mikkonen, Elizabeth Manias, Anne Marie Rafferty, Bryony Dean Franklin, Marja Härkänen

**Affiliations:** ^1^ Department of Nursing Science University of Eastern Finland Kuopio Finland; ^2^ School of Pharmacy, University of Eastern Finland Kuopio Finland; ^3^ Department of Environmental and Biological Sciences University of Eastern Finland Kuopio Finland; ^4^ School of Nursing and Midwifery Monash University Melbourne Australia; ^5^ Florence Nightingale Faculty of Nursing, Midwifery & Palliative Care King's College London London UK; ^6^ School of Pharmacy University College London and NIHR North West London Patient Safety Research Collaboration London UK; ^7^ Research Centre for Nursing Science and Social and Health Management Kuopio University Hospital, Wellbeing Services County of North Savo Kuopio Finland

**Keywords:** cross‐sectional, incident reporting, patient safety, survey

## Abstract

**Aim:**

To investigate nurses' and other users' perceptions and knowledge regarding patient safety incident reporting software and incident reporting.

**Design:**

A cross‐sectional online survey.

**Methods:**

The survey, ‘The Users' Perceptions of Patient Safety Incident Reporting Software’, was developed and used for data collection January–February 2024. We aimed to invite all potential users of reporting software in two wellbeing service counties in Finland to participate in the survey. Potential users (reporters/handlers/others) were nurses, other health professionals and employees. Satisfaction was classified as dissatisfied, neutral, or satisfied. The association between overall satisfaction and demographics was tested using cross‐tabulation and a Chi‐square test.

**Results:**

The completion rate was 54% (*n* = 755). Some respondents (*n* = 25) had never used reporting software, most often due to no perceived need to report, although their average work experience was 15 years. Of other respondents (*n* = 730), mostly nurses (*n* = 432), under half agreed that the software was quick to use and easy to navigate. The biggest dissatisfaction was with the report processing features. Over a fifth did not trust that reporting was anonymous. Training and frequency of using the software were associated with overall satisfaction.

**Conclusion:**

Reporting software has not reached its full potential and needs development. Report handling is essential for shared learning; however, the processing features require the most improvements. Users' perceptions must be considered when developing reporting software and processes.

**Impact:**

Incident reporting software usability is central to reporting, but nurses' and other users' perceptions of software are poorly understood. This survey shows weaknesses in reporting software and emphasizes the importance of training. The survey can contribute to paying more attention to organizing training, getting users to participate in software development, and deepening knowledge of issues in reporting software. Making the needed improvements could improve patient safety.

**Reporting Method:**

The STROBE Checklist (Supplement‐S1).

**Patient or Public Contribution:**

No Patient or Public Contribution.

**What does this paper contribute to the wider global clinical community?**

- Potential users of patient safety incident reporting software must be trained not just to report incidents but also to use reporting software. Some potential software users, reporters, and report handlers, such as nurses and managers with extensive work experience, have never used reporting software.
- Shared learning is the main point in incident reporting, and therefore, reporting software should include genuinely trusted and effective processing options. However, the most dissatisfaction was related to the processing of the reports, and many respondents did not trust software anonymity.
- This survey is helpful for organizations and policymakers in developing patient safety incident reporting software. It raises an understanding of incident reporting software shortcomings and how users perceive its functionality.

## 1 INTRODUCTION

Unsafe care affects millions of patients, relatives, and health professionals annually (World Health Organization, 2020a). Over one in ten patients sustains harm during care, causing huge costs (Klazinga & Slawomirski, 2022; Schwendimann et al., 2018; Slawomirski et al., 2017). Although reporting incidents is essential, underreporting patient safety incidents is a real problem (Hamilton et al., 2018; ten Haken et al., 2021), with one potential reason being reporting software‐related (Hamed & Konstantinidis, 2022; Vrbnjak et al., 2016). Investigating potential incident reporting software users' perspectives, the biggest occupational group being nurses (World Health Organization, 2020b), regarding incident reporting and reporting software is essential to considering further incident reporting development.

## 2 BACKGROUND

Incident reporting is often conducted via electronic software (World Health Organization, 2020a). However, reporting systems have been identified as inadequate (Hamed & Konstantinidis, 2022) and ineffective (Vrbnjak et al., 2016). Brunsveld‐Reinders et al. (2016) found none of the 23 incident reporting systems examined in critical care fulfilled the World Health Organization (WHO) Draft Guidelines for Adverse Event Reporting and Learning Systems checklist requirements. Although the WHO's guideline is from 2005, it is one of the few guidelines for developing reporting systems (Brunsveld‐Reinders et al., 2016). Most systems do not even reach these standards, but today's IT programs should be expected to do much more in 2005. Software development continues, but only a few have been evaluated from the users' perspectives.

Theoretical models have enabled explorations of potential users' behaviour toward and acceptance of different technologies (Rahimi et al., 2018). The Unified Theory of Acceptance and Use of Technology (UTAUT) explains health professionals' acceptance and intention to use technology in healthcare (AlQudah et al., 2021). In the UTAUT model, one of the four determinants focuses on how potential users' expectations of the ease of usability of software are associated with the real use of software (Venkatesh et al., 2003).

**FIGURE 1:**
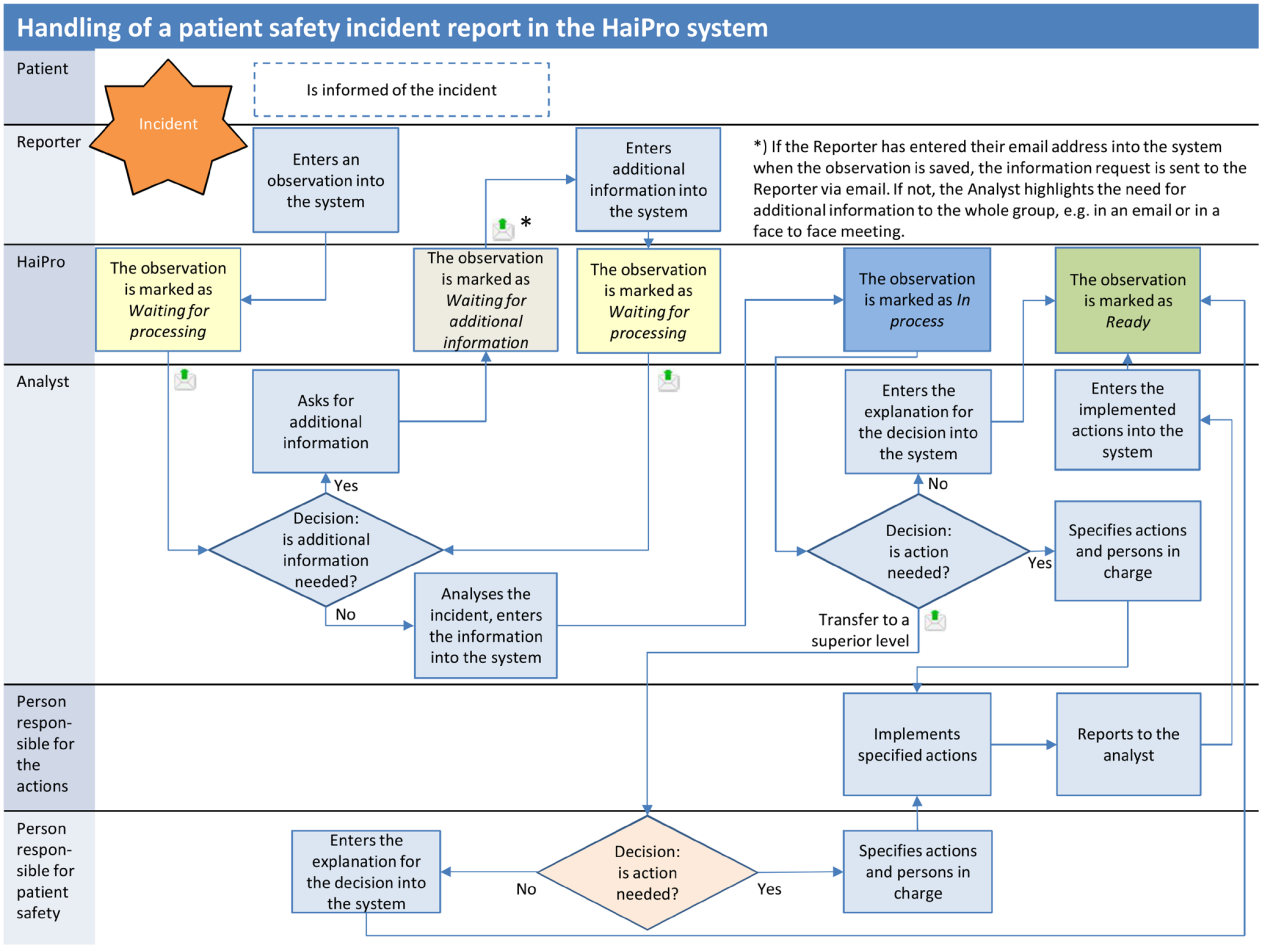
Handling of a patient safety incident report in the HaiPro system (Awanic, 2024).

Software usability is central to incident reporting, but health professionals' perceptions of reporting software are poorly understood and studied (Koskiniemi et al., 2024). In Finland, health professionals and other employees (for example, secretaries) in public healthcare are expected to report patient safety incidents. Other users (for example, patient safety managers) use the software, for example, to monitor care quality. Reporting is voluntary and can be made anonymously. Reports handlers are often unit managers who receive the report and decide on possible further actions. The two most used electronic patient safety incident reporting software in Finland are HaiPro (Figure 1); (Awanic, 2024) and Laatuportti (Qreform, 2024).

The research is topical; content and technical reform of Finland's incident reporting system will be completed by 2025 (Ministry of Social Affairs and Health, 2022). The target is to standardize reporting. In the future, compilations of incident reports may have to be reported publicly. Incident reporting is needed to monitor the quality of care and, most importantly, to be a tool to improve the quality of care and client and patient safety. Understanding reporting software usability from users' perspectives enables software development to better meet practical needs.

## 3 THE STUDY

### 3.1 Aim

The aim of this survey is to investigate nurses' and other users' perceptions and knowledge regarding patient safety incident reporting software and reporting in Finland.

## 4 METHODS

### 4.1 Design

A cross‐sectional design was undertaken, comprising an online survey.

### 4.2 Setting

The setting was two wellbeing service counties in Finland, one using HaiPro (Awanic, 2024) and another Laatuportti (Qreform, 2024). Wellbeing service counties (*n* = 21) oversee organizing public social and healthcare services: specialized healthcare (for example, recovery rooms and outpatient clinics), primary healthcare (for example, oral healthcare and health centres) and social services (for example, home care, and housing services [The Ministry of Social Affairs and Health, 2024]). All units, including one of Finland's five university hospitals, as part of another well‐being service county, were included.

### 4.3 Sample

We aimed to invite all potential users of reporting software (for example, health professionals, managers, safety experts, secretaries, and social service professionals) in two wellbeing service counties (*n* = 27,700) in Finland to participate in the survey.

### 4.4 Survey development

**FIGURE 2:**
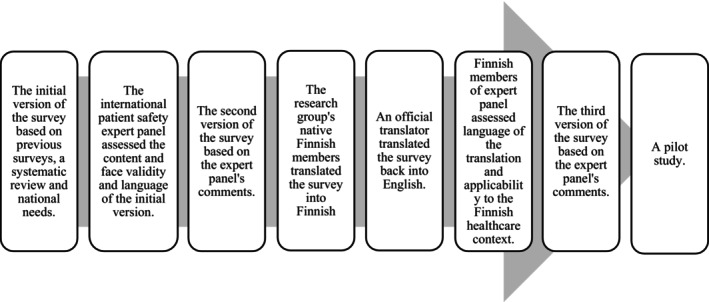
Phases of the users’ perceptions of patient safety incident reporting software (uppsirs) survey development.

The Users' Perceptions of Patient Safety Incident Reporting Software (UPPSIRS) survey was developed for this study following (DeVellis's 2017) tool development phases in 2023 (Figure 2). First, the survey was formed in English based on previous questionnaires (Abu Alrub et al., 2022; Braithwaite et al., 2008; Kuo et al., 2012), a systematic review (Koskiniemi et al., 2024), and designed to reflect current national interest (Ministry of Social Affairs and Health, 2022).

An international multidisciplinary expert panel assessed the initial survey's content and face validity. The expert panel members (*n* = 12) were patient safety specialists, including physicians, professors, main users of incident reporting software, pharmacists, and patient and medication safety officers from Australia (*n* = 4) and Finland (*n* = 8). The number of experts and use of a 4‐point Likert scale for the item rating (1 = not relevant/not clear, 2 = somewhat relevant/somewhat clear, 3 = quite relevant/quite clear, 4 = highly relevant/very clear) was chosen based on Lynn's,1986 recommendations. The content validity index for each item (I‐CVI) was calculated by summarizing the number of experts who scored 3 or 4 and dividing the result by the total number of experts. The calculated I‐CVI range was from 0.64 to 1.00 for relevance and 0.55 to 1 for clarity. Only items with I‐CVI higher than 0.78 for relevance were accepted (Lynn, 1986), which resulted in three items being removed. The overall scale‐CVI/Ave was then 0.91. Almost all items' clarity was revised. The face validity was good; the expert panel agreed that the survey measured users' perceptions of the functionality of the reporting software well.

The research group (*n* = 3) translated the resulting version into Finnish. An official translator back‐translated into English to verify the back‐translation's conformity to the original version. The expert panel's Finnish members (*n* = 6) assessed the Finnish translation and suitability to the Finnish healthcare context.

A pilot study was conducted in November 2023 with purposive sampling of health professionals (*n* = 6) from the authors' networks. Participants meeting the inclusion criteria were requested to assess the items' clarity and language. Based on the pilot study, only minor revisions to some items' clarity were made, and the order of items was changed. The changes were minor regarding wording, so the pilot study participants were included in the final study sample.

The final UPPSIRS survey included background (*n* = 10), open‐ended questions (*n* = 7), and structured items (*n* = 23). The respondents were asked to indicate whether they were reporters and/or reports handlers or other users. Some items were the same for all respondent groups, and some were specific to specific respondent groups. The survey was divided into categories; the last was ‘Overall’ for all respondent groups, including seven items regarding the software's overall functionality. The survey ended after the background questions if respondents answered that they had never used reporting software. Those respondents were asked to write a brief explanation for not using the software. A five‐point Likert scale was used to measure users' perceptions of incident reporting software and knowledge regarding reporting. Higher ratings meant that respondents rated their knowledge of reporting, or the reporting software features better than if they gave a lower rating. An online survey was created using Webropol version 3.0 (2024).

### 4.5 Data collection

Data were collected in January and February 2024. The organization's contact persons emailed the cover letter and two reminders to unit managers, who were asked to share them within the units. The cover letter included a privacy statement, an announcement of the study, and a link to a video that presented the research's content. In one wellbeing service county, the link to the survey was also added to the organization's intranet. The survey remained open for 4 weeks.

It is unknown whether all unit managers shared the cover letter within units and how many software users the survey's cover letter reached. A completion rate was calculated as follows: N of completed questionnaires/N of questionnaires opened by respondents × 100 (Wilson et al., 2024).

### 4.6 Data analysis

Quantitative data were analysed using R software version 4.2.2. (R Core Team, 2021). To meet the statistical test assumptions, some categories of background variables were combined. Respondents' current work titles were classified into nurses, managers, other social and healthcare professionals, and employees. Work units were reclassified into primary and specialized healthcare units, social service units and administration, support, and rescue service units. Demographic data were presented as frequencies and percentages, and continuous variables (age and years of work experience in social or healthcare) as means with the standard deviation. The five‐point Likert scale was condensed into three categories to align with the assumptions required for conducting a chi‐square test to compare the groups: Fully disagree or disagree (Likert scale values 1 and 2), neither agree nor disagree (value 3), and agree or fully agree (values 4 and 5). The overall satisfaction of the software was formulated by calculating the ‘Overall’ category's responses and their averages. Mean ≤2 meant not satisfied, ≥4 satisfied and >2 but <4 neutral. Overall satisfaction connection to background items was tested using cross‐tabulation and a Chi‐square test.

If a respondent indicated that they had never used electronic reporting software, they were asked to write a brief reason for that. Although this study focused on only the quantitative data, these brief reasons were classified based on their similarities. Other open‐ended questions were not analysed and reported in this article.

### 4.7 Ethical considerations

This study followed the prevailing laws and the ethical principles of research. No personal data were processed. Participation in the survey was voluntary and could be discontinued at any time. Participants were asked for consent to participate, and participants' anonymity was guaranteed. Research with human participants in Finland must comply with the Finnish National Board on Research Integrity TENK guidelines. The Finnish National Board on Research Integrity TENK's (2019) guidelines do not require applying an Ethical Statement for this survey. In addition, the Research Ethics Committee of the University of Eastern Finland confirmed that an ethical review was not required (No 17/2023).

## 5 RESULTS

The online survey reached 1398 potential participants, and 755 responded; the completion rate was, therefore, 54%.

### 5.1 Respondents who have never used electronic incident reporting software

Of 755 respondents, 25 (3.3%) said they had never used such software. The average age was 44 years (95% confidence interval [Cl] 38.0–50.3), and work experience in social or healthcare was 15 years (95% Cl 9.1–20.7). Almost half (*n* = 11) were nurses (for example, registered or practical nurses, oral hygienists), and two were managers. According to the brief open answers, the most common reason was no perceived need to report (*n* = 16). Other reasons were that the respondents were new in the organization or had no training, skills, or knowledge for using the software. They also said they did not have time to report and perceived reporting as somehow difficult. One respondent said that nurses report on their behalf. A further respondent said they stopped reporting because it never led to action.

### 5.2 Background information of the respondents

Of the 730 respondents, 512 were reporters, 143 both handlers and reporters, and 75 reports handlers or other users (Table 1). Respondents were 18 to 70 years old (mean 45.9), and most were female (*n* = 657, 90.0%). Respondents' work experiences in social or healthcare varied from zero to 48 years (mean 18.2). Most worked in residential services for older people (*n* = 123, 16.9%) and were registered nurses, public health nurses or midwives (*n* = 211, 28.0%). Respondents were from organizations that used HaiPro (*n* = 522, 71.5%) or Laatuportti (*n* = 207, 28.4%) incident reporting software. One respondent who used reporting software did not know its name. Over half (*n* = 404, 55.3%) had received training for reporting incidents, and over half (*n* = 393, 53.9%) used software at least once monthly.

**TABLE 1:** Characteristic of respondents (*n* = 730) who had used an electronic incident reporting system.

Characteristics	Mean (*SD*)	*n* (%)
Current role in processing patient safety incidents		
Submitter of reports Submitter and handler of reports Handler of reports Other, please specify		512 (70.1) 143 (19.6) 60 (8.2) 15 (2.1)
Gender		
Female Male I prefer not to say Other		657 (90.0) 61 (8.4) 11 (1.5) 1 (0.1)
Age		
18–29 30–44 45–59 Over 60	45.85 (11.47)	68 (9.3) 246 (33.7) 325 (44.5) 91 (12.5)
Main work unit		
Residential services for older people Specialized inpatient ward Home care Health centre Health centre inpatient wards Specialized outpatient clinic Social welfare services Administration Disability services Oral healthcare Imaging services or radiotherapy Service centre for older people Rehabilitation or special services Operating room or post‐anaesthesia care unit Out‐of‐hours services Psychiatric outpatient and inpatient care Intensive care unit or high‐dependency unit Emergency medical care Student or school healthcare Pharmacy (hospital or ward pharmacy) Maternity, child health or family planning clinic Information, equipment, and support services Others (for example, laboratory services, rescue and security services, or substance abuse treatment services)		123 (16.9) 67 (9.2) 66 (9.0) 60 (8.2) 60 (8.2) 59 (8.1) 42 (5.6) 34 (4.7) 30 (4.1) 28 (3.8) 23 (3.2) 20 (2.7) 18 (2.5) 15 (2.1) 14 (1.9) 13 (1.8) 11 (1.5) 10 (1.4) 9 (1.2) 9 (1.2) 7 (1.0) 4 (0.6) 8 (1.1)
Current job title		
Registered nurse, public health nurse or midwife Practical nurse or nursing assistant Nurse manager Counsellor or assistant (e.g., a care assistant or disability counsellor) Rehabilitation or special worker Radiology nurse or clinical laboratory scientist Manager (Not specified whose) Expert (e.g., Patient safety expert) or clinical nursing expert Secretary Dental hygienist Physician manager Dentist Social care or disability service manager Social worker Physician Pharmacist Paramedic Service coordinator or social counsellor Others (for example, psychologists, research coordinators, biologists, health and safety representatives, project directors, managers, coordinators, workers, or clinical nursing or medicine educators) Not known		208 (28.5) 180 (24.7) 113 (15.5) 35 (4.8) 23 (3.1) 21 (2.9) 17 (2.3) 17 (2.3) 15 (2.1) 13 (1.8) 13 (1.8) 12 (1.6) 12 (1.6) 10 (1.4) 8 (1.1) 8 (1.1) 7 (1.0) 6 (0.8) 11 (1.5) 1 (0.1)
Work experience in social or healthcare (years)		
Under one to 2 3–4 5–10 Over 10	18.21 (10.86)	34 (4.7) 40 (5.5) 149 (20.4) 507 (69.4)
Working in an organization using electronic patient safety incident reporting software (years)		
Under one 1–2 3–4 5–10 Over 10 Not known		13 (1.8) 50 (6.8) 75 (10.3) 211 (28.9) 378 (51.8) 3 (0.4)
Used patient safety incident reporting software in the organization		
HaiPro Laatuportti Not known		522 (71.5) 207 (28.4) 1 (0.1)
Received training on the use of currently used reporting software		
No No or not sure Received self‐learning material but did not study it, and/or other teaching was offered but did not attend.		308 (42.2) 18 (2.5)
Yes A colleague or someone else taught or participated in general training. Studied using self‐learning material. Studied self‐learning material, and additionally, a colleague or someone else taught or participated in general training.		204 (27.9) 121 (16.6) 79 (10.8)
Use of the electronic patient safety incident reporting software during the past 12 months		
Daily Weekly Couple times per month Once per month Once every six months Once per year Have not used the system in the past 12 months		10 (1.4) 126 (17.3) 137 (18.8) 120 (16.4) 193 (26.4) 75 (10.3) 69 (9.4)

### 5.3 Respondents' perceptions and knowledge of reporting software

Respondents reported that they know how to find the software on a computer or a mobile device (fully agree or agree 92.7%; Table 2), what kinds of incidents should be reported (fully agree or agree 85.3%), and how to make a report via the software (fully agree or agree 89.1%). Respondents reported that a computer or a mobile device is available when they need to report incidents (fully agree or agree 86.9%). They were satisfied with how the software alerts about missing information while saving the incident report (fully agree or agree 89.9%). Even though most (fully agree or agree 76.7%) were satisfied that it is possible to report anonymously, over half (fully agree or agree 51.5%) said the reporter's and patient's personal data should be given when reporting incidents, at least serious ones.

**TABLE 2:** Users' (*n* = 730) perceptions about reporting software and their own knowledge about reporting.

Category (respondents who answered to a category's items)	Items	Fully disagree or disagree *n* (%)	Neither agree nor disagree *n* (%)	Fully agree or agree *n* (%)
Knowledge (reporters)	1. I know how to find the electronic system for reporting patient safety incidents on my computer and/or from mobile devices if my organization uses a mobile app for reporting.	27 (4.1)	21 (3.2)	604 (92.7)
	2. I know what kinds of patient safety incidents must be reported to the electronic system.	49 (7.6)	46 (7.1)	553 (85.3)
	3. I know how to report patient safety incidents in the electronic system.	32 (5.0)	38 (5.9)	575 (89.1)
Reporting (reporters)	4. Computers or mobile devices are always available when I must fill out an electronic patient safety incident report.	56 (8.6)	29 (4.5)	566 (86.9)
	5. I can follow the handling process of all the incidents I have reported with sufficient accuracy.	124 (19.1)	186 (28.7)	338 (52.2)
	6. I can receive timely feedback after reporting a patient safety incident in the reporting system.	169 (26.4)	235 (36.7)	237 (36.9)
	7. I am satisfied that the reporting system alerts you of erroneous or missing data when saving a patient safety incident report.	12 (1.8)	54 (8.3)	586 (89.9)
Fields and classifications (reporters, handlers, other users)	8. The fields filled out in the incident reporting form are suitable for reporting patient safety incidents.	146 (20.2)	159 (21.9)	419 (57.9)
	9. The pre‐determined response alternatives in the electronic form are clear.	169 (23.4)	157 (21.7)	397 (54.9)
	10. The pre‐determined response alternatives on the electronic form are sufficiently comprehensive for reporting patient safety incidents.	215 (29.6)	180 (24.8)	331 (45.6)
Anonymity (reporters, handlers, other users)	11. I am satisfied that patient safety incidents are reported without patients' personal data.	60 (8.3)	109 (15.0)	556 (76.7)
	12. I trust that the anonymity of those submitting reports is ensured when they report incidents using the electronic reporting system.	161 (22.1)	167 (23.0)	399 (54.9)
	13. The reporter's and patient's personal data should be given when reporting incidents, at least serious ones.	153 (21.3)	196 (27.2)	370 (51.5)
Processing of reports (handlers, other users)	14. Assessing the severity of patient safety incidents is easy based on pre‐determined response alternatives.	62 (28.6)	57 (26.3)	98 (45.1)
	15. The current electronic system for reporting patient safety incidents includes any features necessary for processing reports.	68 (31.3)	57 (26.3)	92 (42.4)
	16. The electronic patient safety incident reports are always automatically referred to the correct person.	102 (47.2)	46 (21.3)	68 (31.5)
Overall (reporters, handlers, other users)	17. The system for reporting patient safety incidents works steadily and does not crash.	49 (6.7)	132 (18.2)	546 (75.1)
	18. The electronic system for reporting patient safety incidents is easy to navigate.	204 (28.2)	167 (23.1)	353 (48.7)
	19. The font and font size used in the reporting system are easy to read.	52 (7.2)	112 (15.4)	562 (77.4)
	20. The electronic system for reporting patient safety incidents is technically simple to use.	141 (19.5)	95 (13.1)	489 (67.4)
	21. The electronic system for reporting patient safety incidents is quick to use	220 (30.5)	146 (20.3)	354 (49.2)
	22. The system for reporting patient safety incidents enables reporting any information relevant to the patient safety incident.	140 (19.4)	175 (24.3)	406 (56.3)
	23. Overall, I am satisfied with the system for reporting patient safety incidents.	121 (16.7)	153 (21.2)	449 (62.1)

Under a third of the respondents reported that the reports reach the correct handler well (fully agree or agree 31.5%), and under half of them said the software includes all necessary features for processing reports (fully agree or agree 42.4%). Under half agreed that the software was quick to use (fully agree or agree 49.2%) or easy to navigate (fully agree or agree 48.7%), and the pre‐determined response alternatives on the form were sufficiently comprehensive (fully agree or agree 45.6%). In addition, under half reported that they could get timely feedback about the report made (fully agree or agree 36.9%).

### 5.4 Overall satisfaction with reporting software

Most respondents' (*n* = 502, 68.8%) overall satisfaction was good. Training received and how often respondents used incident reporting software had a statistically significant association with overall satisfaction with the software (Table 3). The more often respondents used software, the more satisfied they were with the overall features of the software. The result was like the training; if respondents had had training in using reporting software, they would have been more satisfied with the overall features of the software. Other background variables did not have a statistically significant association with overall satisfaction.

**TABLE 3:** Background variables connection to Overall satisfaction with electronic incident reporting software.

	Dissatisfied* *n* (%)	Neutral* *n* (%)	Satisfied* *n* (%)	Total *n* (%)	*p*
Current role in processing patient safety incidents					
Submitter of reports Submitter and handler of reports Handler of reports and others Total	41 (8.0) 9 (6.3) 7 (9.3) 57	125 (24.4) 31 (21.7) 15 (20.0) 171	346 (67.6) 103 (72.0) 53 (70.7) 502	512 (70.1) 143 (19.6) 75 (10.3) 730 (100.0)	.771
Age					
18–29 30–44 45–59 Over 60 Total	5 (3.4) 17 (6.9) 27 (8.3) 8 (8.8) 57	13 (19.1) 65 (26.4) 72 (22.2) 21 (23.1) 171	50 (73.5) 164 (66.7) 226 (69.5) 62 (68.1) 502	68 (9.3) 246 (33.7) 325 (44.5) 91 (12.5) 730 (100.0)	.855
Current work title					
Nurse or nursing staff Manager Other social and healthcare professional Other employee Total	36 (8.3) 12 (7.7) 8 (10.0) 1 (1.6) 57	96 (22.2) 33 (21.3) 22 (27.5) 20 (32.3) 171	300 (69.5) 110 (71.0) 50 (62.5) 41 (66.1) 501	432 (59.3) 155 (21.2) 80 (11.0) 62 (8.5) 729 (100.0)	.347
Current working area					
Social services Specialized healthcare Primary healthcare Administration, support service or rescue service Total	12 (4.3) 21 (10.1) 20 (10.1) 4 (9.3) 57	61 (21.7) 47 (22.6) 51 (25.8) 12 (27.9) 171	208 (74.0) 140 (67.3) 127 (64.1) 27 (62.8) 50	281 (38.5) 208 (28.5) 198 (27.1) 43 (5.9) 730 (100.0)	.162
Working in an organization using electronic patient safety incident reporting software (years)					
Under 5 years 5–10 Over 10 Total	11 (8.0) 15 (7.1) 31 (8.2) 57	34 (24.6) 57 (27.0) 80 (21.2) 171	93 (67.4) 139 (65.9) 267 (70.6) 499	138 (19.0) 211 (29.0) 378 (52.0) 727 (100.0)	.599
Training on the use of currently used reporting software					
No Yes Total	27 (8.3) 30 (7.4) 57	90 (27.6) 81 (20.1) 171	209 (64.1) 293 (72.5) 502	326 (44.7) 404 (55.3) 730 (100.0)	.027
Use of the electronic patient safety incident reporting software during the past 12 months					
Daily or weekly Couple times per month Once per month Once every six months Once per year Have not used the system in the past 12 months Total	8 (5.9) 5 (3.6) 13 (10.8) 13 (6.8) 8 (10.7) 10 (15.5) 57	33 (24.3) 26 (19.0) 19 (15.8) 52 (26.9) 20 (26.7) 21 (30.4) 171	95 (69.9) 106 (77.4) 88 (73.4) 128 (66.3) 47 (62.6) 38 (55.1) 502	136 (18.6) 137 (18.8) 120 (16.4) 193 (26.4) 75 (10.3) 69 (9.5) 730 (100.0)	.015

*Note*: *Dissatisfies = fully disagree or disagree (Likert scale values 1 and 2), neutral = neither agree nor disagree (value 3) and satisfied = agree or fully agree (values 4 and 5).

## 6 DISCUSSION

This study increases understanding of electronic patient safety incident reporting software users' (reporters, reports handlers, and other software users) perceptions of their reporting knowledge and the software's functionality. The key findings are that respondents highly evaluated their knowledge of reporting, but some software features are dysfunctional from many respondents' perspectives. Over a fifth said that incident reporting software is not easy and quick to use. The respondents were more satisfied with overall software features if they had been trained to use the software or if they used the software often. Although most respondents support anonymous reporting, over a fifth do not trust that reporting is anonymous, even if reporters decide to report anonymously. Over half said severe incidents should be reported with the patient and reporter's name.

Respondents' perceptions of incident reporting and reporting software are essential to consider. Patient safety incidents are extremely under‐reported (Hamilton et al., 2018; ten Haken et al., 2021), and reporting software is one reason for non‐reporting (Hamed & Konstantinidis, 2022; Vrbnjak et al., 2016). In a previous study, the perceived simplicity of use greatly influenced dental health professionals' intention to use a current reporting system (Al‐Rayes et al., 2020). In addition, in the UTAUT model, expectations of needed effort are associated with the intention to use technologies (Venkatesh et al., 2003). In this study, under half agreed that the software is quick to use or easy to navigate. Many respondents said that the pre‐determined response alternatives on the electronic form are not sufficiently comprehensive for reporting patient safety incidents. This can lead to incomplete reporting when a reporter interprets an incident as not needing to be reported because it does not fit the pre‐determined response alternatives on the electronic form (Waters et al., 2012).

The software features for processing the reports received the most unsatisfied assessment. Almost a third of the report handlers in this study perceived that the software did not include all the necessary features to process the reports. The reports should reach the correct handler based on the information reporters fill into the software. Nevertheless, almost half of the handlers said the reports go to the wrong handlers. Reporting does not improve client and patient safety; the shared learning after reporting is most important. Nevertheless, much more has been invested in reporting than the handling process (World Health Organization, 2020a), and utilizing valuable information received via incident reporting is not at the level it should be (Liukka et al., 2019).

Training and how often respondents used reporting software were associated with overall satisfaction with the reporting software. The respondents were more satisfied with the overall features of the software if they had been trained to use it or if they used it often. In this study, almost half of respondents had not received training for using current software. The lack of training aligns with Hamed and Konstantinidis's (2022) study, which states that nurses are not always trained in incident reporting. Of all respondents, 3.3% said they had never used reporting software. Over half of them said they did not need the software even though their average work experience in social or healthcare was 15 years. Lack of knowledge is one reason for non‐reporting among potential users in this study and health professionals in previous research (Hamed & Konstantinidis, 2022; Vrbnjak et al., 2016). Thus, focusing on training for all potential incident reporters is essential, as it could raise reporting rates.

Although most respondents were satisfied with the possibility of reporting anonymously, over half responded that serious incidents should be reported with reporter and patient identification data. This contrasts with previous studies where the lack of anonymous reporting has been reported as a reporting barrier (Hamed & Konstantinidis, 2022). In the future, it would be valuable to identify differences in opinions between professional groups. In Finland, the current client and patient strategy states that the reporting obligation will be assessed in the future. Currently, reporting is voluntary (The Ministry of Social Affairs and Health, 2022).

If users do not report patient safety incidents due to inadequate reporting software, incidents can repeat time after time. Improving reporting software is an act for client and patient safety. In the future, end‐users must be part of developing health technologies and not just participate in the testing phases. Electronic incident reporting software faces the same difficulties in fulfilling its purpose around the world (Brunsveld‐Reinders et al., 2016).

### 6.1 Strengths and limitations

Based on our knowledge, this study was the first to provide an overview of the usability of incident reporting software from the users' viewpoint in Finland. This study's strength is the development process of the UPPSIRS questionnaire. The process followed DeVellis's (2017) steps and was developed based on three previous surveys (Abu Alrub et al., 2022; Braithwaite et al., 2008; Kuo et al., 2012), a systematic review (Koskiniemi et al., 2024), and national needs (Ministry of Social Affairs and Health, 2022). An international expert panel assessed the questionnaire's face and content validity.

The completion rate was good, 54%, which might reflect that nurses and other users genuinely perceive reporting software as an essential part of the incident reporting process. However, the response rate could not be calculated because the number of potential software users with whom unit managers shared in the survey is unknown. The sample size was relatively large, and the total missing data was low, with only 120 responses to the survey's items. The highest percentage of missing data in one item was 1.95% (item 6). All employees in wellbeing service counties can report patient safety incidents and were able to participate in this study, but most of the respondents were nurses; it must be noted that some occupational groups were poorly represented.

### 6.2 Recommendation for policy and practice

This study shows weaknesses in incident reporting software and processes and emphasizes the importance of user training in the actual use of reporting software. Those responsible for developing reporting software should make sure that incident reporting software includes all the necessary features for handling reports. The reports should be directed automatically to the right handlers based on information reporters fill into the software, which does not materialize now. It is also justified to think about whether the reporting must be anonymous. Many participants in this study did not trust that anonymous reporting is truly anonymous. On the other hand, over half agreed that at least severe incidents should be reported with the patient and reporter's names. This would facilitate the support of all incident participants.

During software reforms, end‐users' perceptions must be taken into account. Moreover, they must be involved directly in the development process, not just in the piloting phase, straight from the beginning. It would be useful to involve individuals who are not interested in reporting, who have never used incident reporting software and who are dissatisfied with current software in addition to active software users.

## 7 CONCLUSIONS

Nurses and other users were satisfied with their knowledge regarding incident reporting but reported development needs, especially in features regarding processing reports. Reports do not appear to reach the right handler, which increases the reports' processing time. Quite often, the reporting software is perceived as slow to use, hard to navigate, and lacking sufficiently comprehensive pre‐determined response alternatives for reporting patient safety incidents. Many respondents do not trust the anonymity of the software but think that severe incidents should be reported with a patient and reporter's name. Training and use of reporting software were associated with overall satisfaction with the software. Organizations need to ensure that all potential incident reporting software users have been trained for its use. The next step will be to analyse the open‐ended answers to the collected data for this survey to obtain in‐depth and complementary information on the subject.

## AUTHOR CONTRIBUTIONS

SK, TS, KHA, SM, EM, AMR, BDF & MH: Made substantial contributions to conception and design, or acquisition of data, or analysis and interpretation of data; SK, TS, KHA, SM, EM, AMR, BDF & MH: Involved in drafting the manuscript or revising it critically for important intellectual content; SK, TS, KHA, SM, EM, AMR, BDF & MH: Given final approval of the version to be published. Each author should have participated sufficiently in the work to take public responsibility for appropriate portions of the content; SK, TS, KHA & MH: Agreed to be accountable for all aspects of the work in ensuring that questions related to the accuracy or integrity of any part of the work are appropriately investigated and resolved.

## FUNDING INFORMATION

This work was supported by the Research Council of Finland (grant number 353503). BDF is supported via the National Institute for Health and Care Research (NIHR) North West London Patient Safety Research Collaboration (PSRC). The views expressed are those of the author(s) and not necessarily those of the NHS, the NIHR, PHE or the Department of Health and Social Care.

## CONFLICT OF INTEREST STATEMENT

None.

## PEER REVIEW

The peer review history for this article is available at https://www.webofscience.com/api/gateway/wos/peer‐review/10.1111/jan.16364.

## Supporting information


Appendix S1.

## Data Availability

The research data is permanently archived without personal data at the Finnish Social Science Data Archive. The Archive may hand over the data for reuse to registered clients for research, teaching, and study after the 1st of January 2026.
